# A Relation-Oriented Model With Global Context Information for Joint Extraction of Overlapping Relations and Entities

**DOI:** 10.3389/fnbot.2022.914705

**Published:** 2022-07-04

**Authors:** Huihui Han, Jian Wang, Xiaowen Wang

**Affiliations:** ^1^Country Computer Integrated Manufacturing System Research Center, College of Electronics and Information Engineering, Tongji University, Shanghai, China; ^2^Tsinghua Shenzhen International Graduate School, Tsinghua University, Shenzhen, China

**Keywords:** joint extraction of entities and relations, multi-label classification, relation extraction, entity recognition, overlapping triples

## Abstract

The entity relation extraction in the form of triples from unstructured text is a key step for self-learning knowledge graph construction. Two main methods have been proposed to extract relation triples, namely, the pipeline method and the joint learning approach. However, these models do not deal with the overlapping relation problem well. To overcome this challenge, we present a relation-oriented model with global context information for joint entity relation extraction, namely, ROMGCJE, which is an encoder–decoder model. The encoder layer aims to build long-term dependencies among words and capture rich global context representation. Besides, the relation-aware attention mechanism is applied to make use of the relation information to guide the entity detection. The decoder part consists of a multi-relation classifier for the relation classification task, and an improved long short-term memory for the entity recognition task. Finally, the minimum risk training mechanism is introduced to jointly train the model to generate final relation triples. Comprehensive experiments conducted on two public datasets, NYT and WebNLG, show that our model can effectively extract overlapping relation triples and outperforms the current state-of-the-art methods.

## Introduction

Relation extraction (RE) is a significant task for constructing self-learning knowledge graphs (KGs), which are graph-structured facts usually in the form of triple. The relationship between an entity pair (*e*_*i*_, *e*_*j*_) can be formalized as a relational triple (*e*_*i*_, *r*_*ij*_, *e*_*j*_), where *e*_*i*_ and *e*_*j*_ represent the head and tail entity, respectively, and *r*_*ij*_ denotes a specific type of relationship connecting *e*_*i*_ to *e*_*j*_. The objective of RE is to extract relation triples from unstructured text without human intervention based on predefined entity and relation categories. The well-structured nature of relational triples is well-suited to develop KG, which is widely used in intelligent robot, intelligent recommendation, intelligent furniture, and so on. In particular, products with question-and-answer functions such as chatbots (e.g., Siri, Microsoft Xiaoice, Tmall Genie, Xiaomi speakers, etc.) require the support of large-scale KG. However, the RE model with bad performance will lead to incomplete KG (Wu and Luo, 2020; Liu et al., 2021). Therefore, it is essential to construct effective RE models to extract all triples from texts.

Conventional RE task is based on pipeline methods in which the named entity recognition (NER) part is first applied to recognize entities in a sequence (Liu et al., 2010; Vazquez et al., 2011; Skeppstedt et al., 2014), and then a relation classification (RC) part is used to assign the predefined relation types to these candidate entity pairs (Santos et al., 2015; Zhang et al., 2018; Ren et al., 2022). After these two sequential steps, triples are finally extracted. Although such a structure makes the task simple to conduct, it ignores the hidden interdependency and error propagation between these two subtasks, which leads to low accuracy in extracting relation triples. To overcome these problems, joint extraction methods, simultaneously detecting entities together with their relations from unstructured texts, are proposed.

As shown in Table 1, sentences are generally classified into three types according to the overlapping degree of triples (Zeng et al., 2018), namely, (1) normal: there is no entity belonging to two or more different triples simultaneously in a sentence; (2) entity pair overlap (EPO): there is entity pair (*e*_*i*_, *e*_*j*_) sharing two or more different relations, i.e., $\left(e_{i},\;r_{ij}^{1},\;e_{j}\right)$, $\left(e_{i},r_{ij}^{2},\;e_{j}\right),\;\cdots \;,\;\left(e_{i},\;r_{ij}^{n},\;e_{j}\right)$ in a sentence. (3) single entity overlap (SEO): there is an entity *e*_*i*_ that has two or more relations with different entities in a sentence, i.e., (*e*_*i*_, *r*_*ij*_, *e*_*j*_), (*e*_*i*_, *r*_*ik*_, *e*_*k*_), ⋯, (*e*_*i*_, *r*_*iq*_, *e*_*q*_). Due to the existence of complex overlapping relations (e.g., SEO or EPO), the joint extraction methods still face great challenges in extracting relation triples.

**Table 1:** Examples of sentence types, normal, EPO, and SEO.

**Type**	**Sentences**	**Relation triples**
Normal	Capitol Hill is in Washington.	< *Capitol Hill, Contains, Washington* >
EPO	Joe Biden is the American president.	< *Joe Biden, Country-President, America* >
		< *Joe Biden, Nationality, America* >
SEO	George lives on Mount Fuji in Japan.	< *George, placelived, Japan* > < *Japan, contains, Mount Fuji* >

Recently, extensive research works have been conducted in joint extraction. These works are divided into two directions based on the extraction order of the triple elements: entity first and relation first. The entity-first method can be formed as (*e*_*i*_, *e*_*j*_) → *r*_*ij*_, which first identifies all entities applying NER techniques, and then assigns relation types to these candidate entity pairs. Yu et al. (2019) proposed a new model ETL–Span, where the head entities were first identified, and then the matching tail entities and relations were recognized by using some joint decoding approaches. Li et al. (2019) developed a novel paradigm by casting the RE task as a multi-turn question answering problem, i.e., the extraction of entities and relations was changed to the task of recognizing answer spans from the context. In this process, the head entity was first extracted, and then the tail entity and the relation were detected.

The relation-first method is formed as *r*_*ij*_, → (*e*_*i*_, *e*_*j*_), where the relation information can be used as the prior knowledge to guide the extraction of semantically related entities. In the CopyRE model (Zeng et al., 2018), the relation was first generated by the decoder with the copy mechanism. Then the copy mechanism was adopted to extract the head entity and tail entity from the source text. However, CopyRE cannot distinguish head and tail entities or predict multi-token entities (e.g., Steven Jobs). To solve these problems, Takanobu et al. (2019) proposed the hierarchical reinforcement learning (HRL) framework, where a high-level reinforcement learning (RL) was used to detect the relations, and a low-level RL was applied to identify the participating entities related to the detected relations.

**Figure 1:**
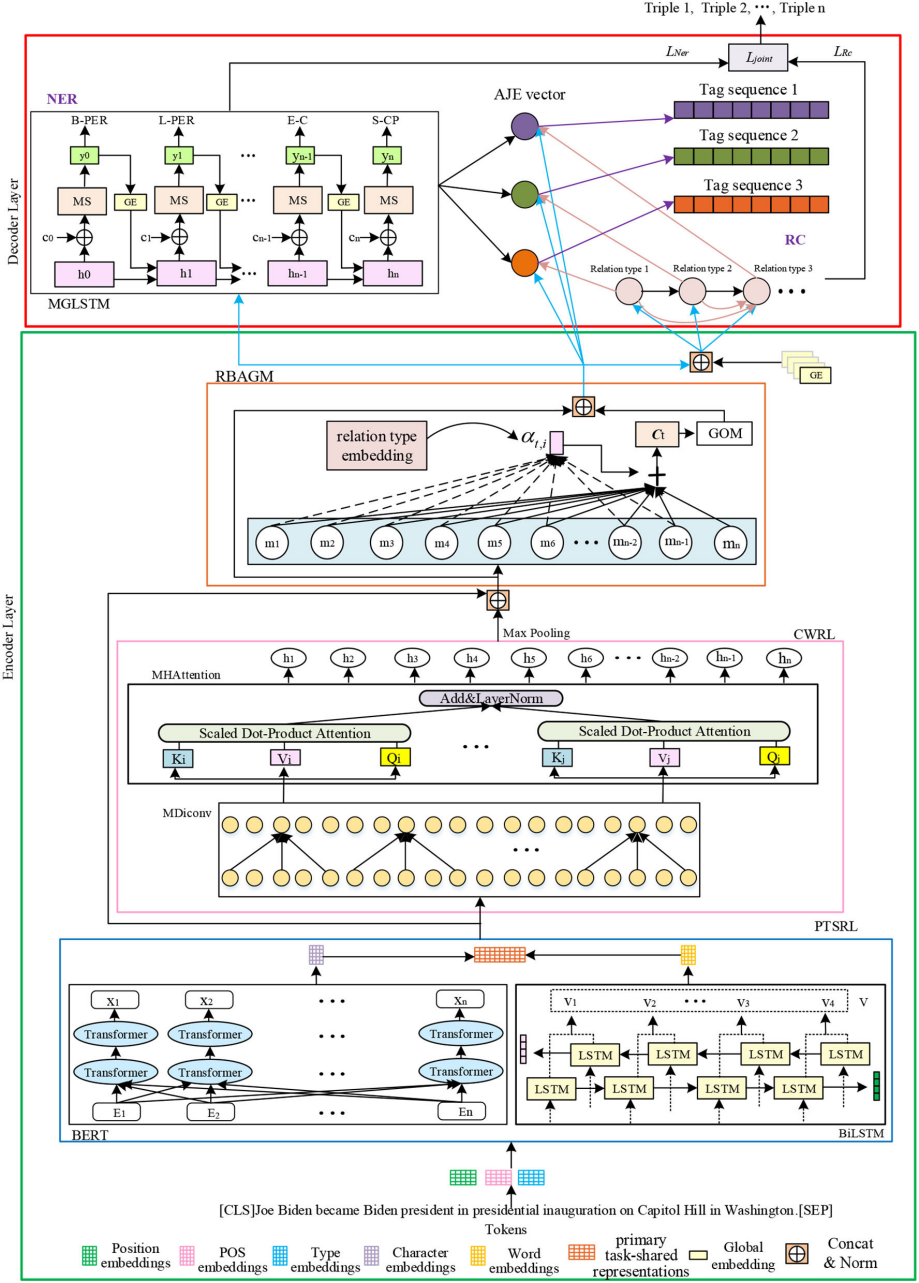
The structure of ROMGCJE. The tokens of sentences are first represented as distributed word representations, which are then fed to the encoder layer containing PTSRL, CWRL, and RBAGM to capture rich contextual information and build long range dependency. Next, MGLSTM and MRC in the decoder layer are applied to perform decoding for NER and RC tasks, respectively. Finally, the MRT mechanism is applied to train ROMGCJE to extract the final relation triples.

Although the above approaches have achieved reasonable performance in extracting relation triples, they all suffer from the same problem, namely, exposure bias. Besides, these models neglect the semantic connections between words in a text. Therefore, the problem of extracting overlapping relations is not overcome. To this end, this study proposes a novel relation-oriented model with global context information for joint extraction (ROMGCJE), which can effectively extract the overlapping relation triples from the unstructured texts. As illustrated in Figure 1, this model is constructed in the encoder–decoder structure. The encoder part consists of a primary task-shared representation layer (PTSRL), a contextual word representation layer (CWRL), and a relation-based attention module with a gating mechanism (RBAGM). First, a tagging scheme (Liu et al., 2019) is applied to convert the joint extraction task to a sequence labeling problem, mapping a sentence $S={\left\{w_{t}\right\}}_{t=1}^{n}$ into a tag sequence $Tag={\left\{Tag_{t}\right\}}_{t=1}^{n}$. Then, the PTSRL, composed of BERT (Devlin et al., 2018) and bidirectional long short-term memory neural network (BiLSTM) (Schuster and Paliwal, 1997), is used to obtain word embedding. Next, the CWRL, made of a multi-head attention module (MHAttention) and multi-layer dilated convolution (MDiconv) module with residual blocks, is proposed to encode words to capture rich global contextual features, build long-range dependency among words, and more fully extract the semantic connections between words. Furthermore, the RBAGM is designed to utilize the relation information to guide the detection of entities. The encoder layer makes the ROMGCJE model jointly express both entities and relations with shared parameters in a single model. The decoder part contains a multiple relation classifier (MRC) (Read et al., 2011) for extracting relations and an improved LSTM (MGLSTM) for detecting entities. Furthermore, an attention module is used to match the corresponding entities in line with the recognized relations. Eventually, the minimum risk training (MRT) mechanism (Sun et al., 2018) is introduced to train the model to make the training process more stable. This approach does not desire any supplementary manual features or natural language processing (NLP) tools compared with feature-based methods. In addition, this new model not only prevents superfluous information from being generated but also tackle overlapping relations effectively.

The contributions of this study are summarized as follows: (1) A novel relation-oriented model with global context information is proposed for joint extraction. The relation-aware attention mechanism is applied to make use of the relation information to guide the entity detection, which can reduce the extraction of redundant entities. (2) This model takes into consideration the rich global contextual information, builds long-range dependency among words, and fully extracts the semantics of the passage in extracting relation triples. (3) Extensive experiments conducted on public datasets (NYT and WebNLG) demonstrate that the proposed model can achieve state-of-the-art performance in extracting overlapping relation triples.

## Related Work

Relation extraction has become progressively critical in KG construction, smart robots, search engine, intelligent question–answering systems, etc. The pipeline method and joint method are the mainstream methods for extracting entities and relations from unstructured texts.

In early works, the pipeline method is intensively investigated, which divides the task into two serial independent subtasks, NER and RC; NER, recognizing the named entities, generally, is defined as a sequence tagging task. There are two research directions, i.e., statistical method (Gong et al., 2019; Alam and Islam, 2020) and neural network method (NNM) (Wei et al., 2019; Zhang et al., 2019). For the statistical machine learning-based method, the conditional random fields (CRF) mechanism is widely used, and the feature engineering and corpora play critical roles in improving extraction accuracy (Dalvi et al., 2011). Neural network method typically uses neural networks, e.g., convolutional neural networks (CNNs) or recurrent neural networks (RNNs), to learn sentence features without tedious feature engineering. There are some classic models, such as BiLSTM + CRF (Huang et al., 2015), deep neural networks +R (Tomori et al., 2016), and so on. In addition, it can achieve outstanding results by combining with the pre-trained language models such as BERT (Dai et al., 2019a). Relation classification, determining the relationship type between two entities, generally, is taken as a multi-label classification task. Zhou et al. (2016) introduced the attention mechanism to determine the contribution of each word to the final results. Zhang et al. (2017) applied the position-aware attention mechanism to refine the information of position embedding. However, the above approaches require preprocessing step NER, which leads to that the errors generated in the NER stage will be propagated to the RC stage (Zeng et al., 2018). Besides, NER task and RC task are realized by different models without joint training, which leads to that the inherent correlation between NER task and RC task is neglected and extensive unrelated entity pairs are generated in the pairing phase (Zhao et al., 2021).

To overcome the limitations of pipeline methods, a number of joint extraction methods have been proposed (Bekoulis et al., 2018). Zheng et al. (2017) presented a novel tagging scheme to convert the extracting task into a sequence labeling problem, which could make use of the inherent correlation between the RC task and the NER task. Nevertheless, this approach can only extract triples from normal sentences where there is no entity belonging to two or more different triples simultaneously. To extract overlapping relation triples, Zeng et al. (2018) put forward an end-to-end model with the copy mechanism, but the NER part heavily depended on word segmentation tools. Bekoulis et al. (2018) proposed a joint training model, where the NER task was realized by the CRF layer and the RC task was taken as a multi-head selection problem. Fu and Ma (2019) put forward a new joint model based on the LSTM and graph convolutional networks (GCNs). However, the above approaches only deal with the SEO problems and fail to solve the EPO problems, since they cannot assign different relation tags to one token.

To overcome the EPO problems, great efforts have been made. Li et al. (2021) proposed a translating decoding schema for joint extraction of entities and relations (TDEER), but this model did not deal with the error accumulation problem. To solve this problem, Wang et al. (2020a) transformed the joint extraction task into a token pair linking (TPLinker) problem, which did not contain any interdependent stages. Although the TPLinker could alleviate the error accumulation problem, processing all token pairs at encoder layers led to high computation complexity in encoding long paragraphs. Chen et al. (2019) proposed a novel architecture, where a BiLSTM classifier was first applied for identifying all possible relations maintained in the text, and then multi-head attention was performed to generate all possible entity pairs sequentially. Eberts and Ulges (2019) developed an attention model for span-based joint entity and relation extraction. The model achieved excellent performance but still suffered from the redundancy problem. Dai et al. (2019b) proposed a tagging scheme to produce *m* tag sequences for a sentence with *m* words, and applied a position-attention mechanism to generate different sentence representations for each query position. The joint extraction task was decomposed into the following two subtasks as in the study by Wang et al. (2020b): Head entity extraction and tail entity extraction, which solved the problem of SEO in the triples. Then the tail entity extraction was divided into three parallel sub-processes to solve the EPO problem of triples. Nevertheless, because of the labeling-once process, the above models always ignore the inner dependency among head entities, tail entities, and relations. Luo et al. (2020) proposed a bidirectional tree tagging scheme to label overlapping triples in text, which promoted the extraction of overlapping relation triples to some extent. Unfortunately, these models failed to extract overlapping relation triples in high accuracy, since they do not take into consideration the underlying contextual information or the semantic relation between words during extracting overlapping relation triples. Accordingly, the overlapping relation problem is not handled. Based on the above analysis, we propose a new relation-oriented model with global context information for the joint extraction of overlapping relations and entities. This model can deal with the overlapping relations effectively, since it can take full advantage of rich global contextual information, build long-range dependency among words, and more fully extract the semantics of the passage in extracting relation triples.

## Model Description

In this section, the task description is first introduced. Then we describe the tagging method and explain how to change the extraction task to a tagging problem. Finally, the new model ROMGCJE is introduced in detail, which can extract overlapping relation triples effectively.

### Task Description

The objective of this new model is to extract all entities together with their relations in the form of triples from unstructured text. The relational triple is formed as (*e*_*i*_, *r*_*ij*_, *e*_*j*_), where *e*_*i*_ ∈ *E, e*_*j*_ ∈ *E, r*_*ij*_ ∈ *R*. Denote *E* and *R* as a set of predefined entities and relations, respectively. Especially, some entities or relations may exist in multiple triples. The entity extraction is taken as a sequence tagging task, where different tags are assigned to different words in the input sequence. The RC task is taken as a multi-label classification task. During the extraction process, entity information and relation information can interact.

### Tagging Method

To overcome the problem of overlapping relations, the Beginning, Inside, End, Outside, and Single (BIEOS) scheme is applied to label entities and relations in sentences. The word's tag contains information about the word's position in the entity, the relation type, and the relation role. The word position information refers to BIEOS. All relation types come from the predefined set of relations, and the numbers “1” and “2” are used to represent the relation role information. “1” represents that the word belongs to the first entity in the triple, while “2” denotes that the word belongs to the second entity in the triple.

**Figure 2:**
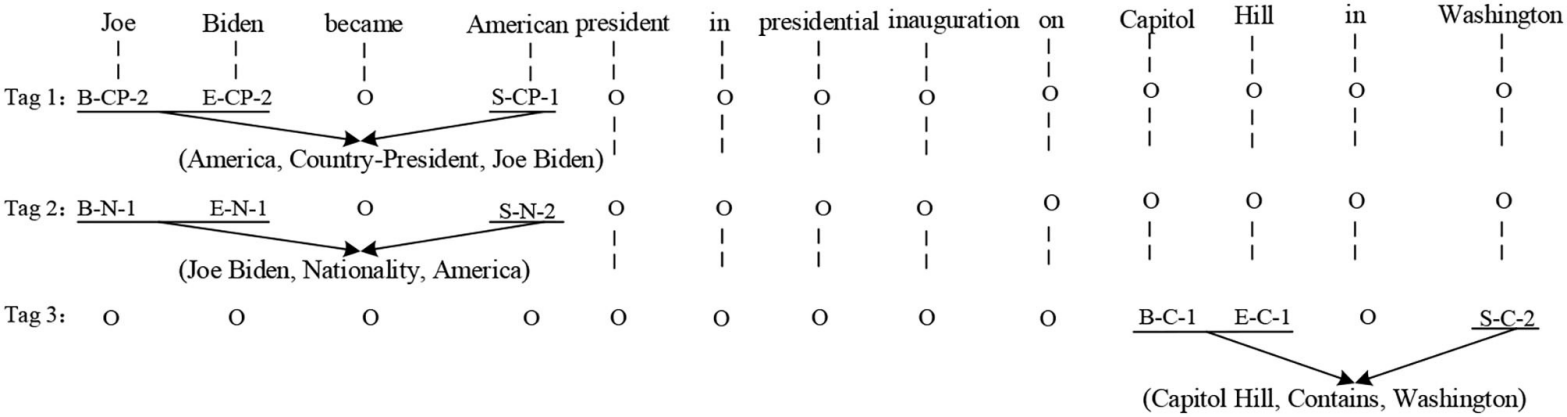
An example of the tagging scheme.

Figure 2 explains how the sentences are tagged by using the BIEOS scheme (Liu et al., 2019). In one sentence, multiple tag sequences are given, and each tag sequence only contains a triple. Therefore, even though there exist overlapping relations in a sentence, entities can be assigned to the right labels. The input sentence contains three triples: {Joe Biden, Country-President, America}, {Joe Biden, Nationality, America}, and {Capitol Hill, Contains, Washington}, where “Country-President,” “Nationality,” and “Contains” are the predefined relation types. The words “Joe,” “Biden,” “America,” “Capitol,” “Hill,” and “Washington” are related to the predefined relation types. For instance, the word “Joe” is the first word of the entity “Joe Biden” and is related to the relation “Country-President,” so its tag is “B-CP-1.” The other entity, “America,” which corresponds to “Joe Biden,” is labeled as “S-CP-1.” Besides, other words irrelevant to the final result are labeled as “‘O.” We combine entities with the same relation type into a triple to get the final result. Specifically, “Joe Biden” and “America” can be combined into a triple whose relation type is “Country-President.” Because the relation role of “Joe Biden” is “2” and “America” is “1,' the final result is {America, Country-President, Joe Biden}. Besides, if a sentence contains two or more triples with the same relation type, we combine every two entities into a triple based on the nearest principle. For example, if the relation type “Country-President” in Figure 2 is replaced by “Contains,” then there will be four entities with the same relation type in the given sentence. “America” is closest to the entity “Joe Biden,” and the “Capitol Hill” is closest to “Washington,” so the final results will be {America, Contains, Joe Biden} and {Capitol Hill, Contains, Washington}.

Evidently, the entities can be used multiple times in the different relation triples. Inspired by Miwa and Bansal (2016), the random start and fine-turned operations are applied to these tag labels during training. Notice that the ground-truth labels are only used during training, whereas the predicted labels are used at inference time.

### Encoder Layer

The encoder layer, composed of PTSRL, CWRL, and RBAGM, is designed to better capture global contextual information, build long-range dependency among words, and fully extract the semantics of text. Note that we add a [CLS] token in front of the sequence and a [SEP] token at the end of the sequence.

#### PTSRL

As illustrated in Figure 1, the PTSRL consists of a BERT pretraining model and a BiLSTM. Given a sentence that consists of *n* words $S={\left\{w_{t}\right\}}_{t=1}^{n}$, where *w*_*t*_ represents the *tth* word, we map each token in the sentence to a real-valued embedding to express its semantic and syntactic meaning through the BERT word embedding layer, and get $V^{w}={\left\{v_{t}^{w}\right\}}_{t=1}^{n}$ by Equation (1),


(1) $$\begin{array}{l} v_{t}^{w}\;=\;BERT\left(word\left(w_{t}\right);w_{t}\right) \end{array}$$

where $v_{t}^{w}\in {\mathbb{R}}^{d}$ represents the *d*-dimensional word vector embedded to the *tth* word in the sentence.

As out of vocabulary word is common for entity, we also augment word representation with character-level information. A BiLSTM network, as illustrated in Figure 1, is applied to obtain the character-level representations $U^{c}={\left\{u_{t}^{c}\right\}}_{t=1}^{n}$ by Equation (2), which effectively captures the morphological information of the word.


(2) $$\begin{array}{l} u_{t}^{c}\;=\;BiLSTM\left(\left[char\left(w_{t}\right);w_{c}\right]\right) \end{array}$$

The primary task-shared representations $X={\left\{x_{t}\right\}}_{t=1}^{n}=\left[v_{t}^{w};u_{t}^{c}\right]$ containing the word-level semantic information are the concatenation of word-level and character-level representation.

#### Contextual Word Representation Layer

In the multi-label classification task, *n*_*T*_ relation types represent *n*_*T*_ classes. Each relation type in one sentence has semantic units that constitute the entire text's semantic meaning. The primary task-shared representations are not enough for encoding the *n*_*T*_ tag sequences in a sentence. Therefore, we design the CWRL, which is composed of the MDiconv module, MHAttention module, and Max-Pooling module, to capture semantic units, build long sequence information dependence, and extract rich global contextual information.

##### MDiconv Module

**Figure 3:**
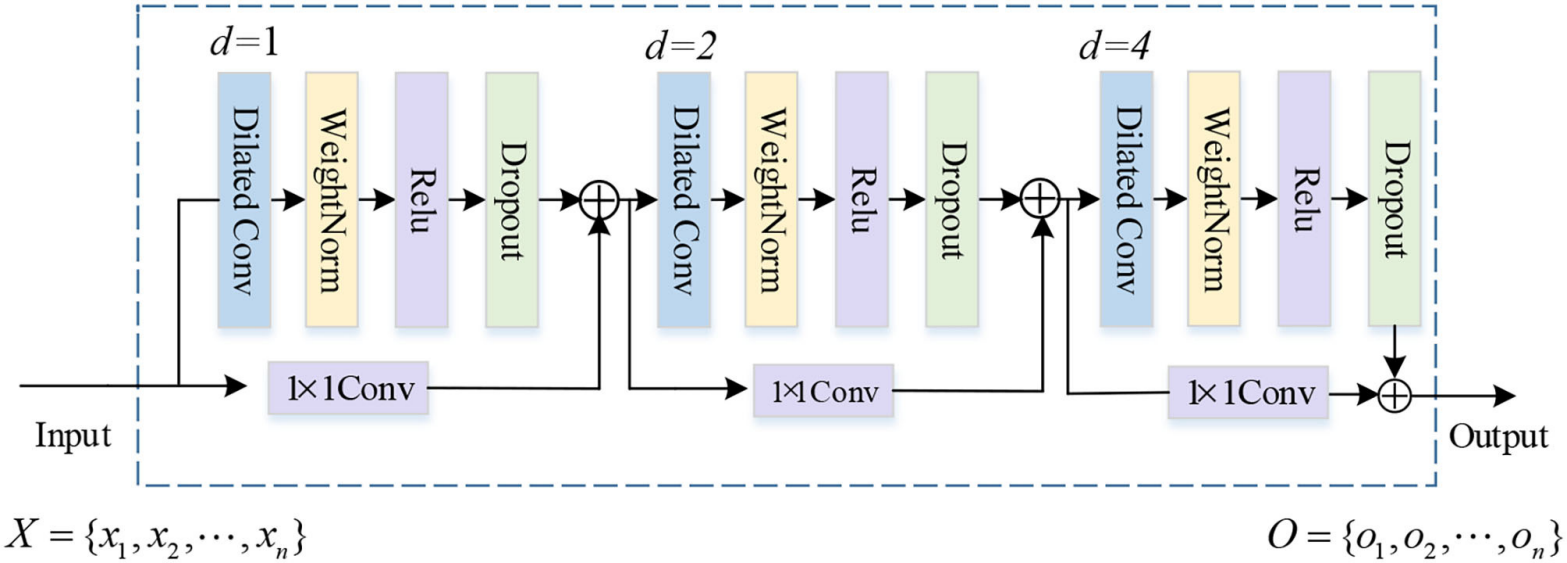
The structure of MDiconv. The dilation factors *d* = 1, 2, and 4, and filter size *k* = 3.

Plenty of research works have proved that the dilated convolution (Diconv) performs well in expanding the receiving field without losing position and semantic information. Inspired by Salimans and Kingma (2016), we design the Mdiconv module. As we all know, the deeper network usually can extract more abstract features and richer semantic information. However, simply increasing the network depth will lead to gradient explosion and gradient disappearance. To deal with this problem, the skip connection is introduced to the Mdiconv module. As shown in Figure 3, there are three residual blocks. Each residual block is composed of the dilated causal convolution, weight normalization, rectified linear unit (ReLU), and spatial dropout for regularization. A 1 × 1 convolution is introduced into each residual block, since there is some difference in dimension between the input and output. After the elementwise addition ⊕ operator, the features are fed to the next residual block, and finally we obtain the final sentence representations $O={\left\{O_{t}\right\}}_{t=1}^{n}$. Because of the introduction of the skip connection, MDiconv exhibits longer memory, which makes it suitable for encoding long sentences containing entities far from each other.

##### MHAttention Module

The multi-head self-attention is applied to capture arbitrary interactions between tokens. It has several merits as follows: (1) Building long-range dependencies by explicitly attending to all positions (2) disambiguating homonyms, and expressing semantic and syntactic patterns greatly. Specifically, the hidden features $H={\left\{h_{t}\right\}}_{t=1}^{n}$ of the MHAttention module are calculated as follows.


(3) $$\begin{array}{l} Q\;=\;OW_{j}^{Q} \end{array}$$

(4) $$\begin{array}{l} K\;=\;OW_{j}^{K} \end{array}$$

(5) $$\begin{array}{l} V\;=\;OW_{j}^{V} \end{array}$$

(6) $$\begin{array}{l} H\;=\;\left(\left[head_{1};\cdots \;;head_{z}\right]\right)W \end{array}$$

(7) $$\begin{array}{l} head_{j}\;=\;softmax\left(\frac{QK^{T}}{\sqrt{d_{k}}}\right)V \end{array}$$

where different linear layers are used in Equations (3)–(5) to map the input *O* into different subspaces by learnable parameters $W_{j}^{Q},\;W_{j}^{K}$, and $W_{j}^{V}$ yielding query *Q*, keys *K*, and values *V*. Then *z* parallel heads are employed to develop interactions in the different parts of channels to generate text representation *H*. The representation *head*_*j*_ of the *jth* head is calculated by a scaled dot-product attention operation in Equation (7).

Then, a residual connection along with layer normalization is applied on *O* and *H* to generate the output hidden features $H={\left\{h_{t}\right\}}_{t=1}^{n}$. Finally, a max-pooling layer is appended on all hidden states of the MHAttention module to capture a sentence-level feature $\partial ={\left\{\partial_{t}\right\}}_{t=\;1}^{n}$.


(8) $$\begin{array}{l} \partial_{t}\;=\;maxp\;\left(h_{t}\right) \end{array}$$

where *maxp* represents the max-pooling operation.

The sentence feature representations *M* is obtained by Equation (9), which encodes the semantic information of its context.


(9) $$\begin{array}{l} M\;=\;{\left\{m_{t}\right\}}_{t=1}^{n}\;=\;Norm\left(\left[X;H;\partial ;\right]\right) \end{array}$$

where *Norm* represents a normalization operation.

#### Relation-Based Attention Module With a Gating Mechanism

The different words in a sentence with the different relations play the different roles. Therefore, relation-based attention (RBA) (Yuan et al., 2020) is applied to help calculate the sentence representations. The attention weight is proportional to the influence of the word at the current decoding time. When considering the relation type *r*_*k*_, the specific context sentence representation *c*_*k*_ is calculated by the weighted sum of the sentence words, defined by Equations (10)–(12).


(10) $$\begin{array}{l} e_{ki}\;=\;tanh\left(W_{h}m_{i}\;+\;W_{s}M+{W_{k}r_{k}\;+\;b}_{attn}\right) \end{array}$$

(11) $$\alpha_{t,\;i}\;=\;\frac{\text{exp}(e_{ti})}{\sum_{i=1}^{n}\text{exp}(e_{ti})}$$

(12) $$\begin{array}{l} c_{k}\;=\;\overset{n}{\underset{i}{\sum }}\alpha_{t,\;i}m_{i} \end{array}$$

where weight matric *W*_*h*_, *W*_*s*_, *and W*_*k*_, and bias vector *b*_*attn*_ are the parameters; *m*_*i*_ denotes the hidden state vector of the *ith* word in the encoder layer; *M* represents the global representation of the sentence; *r*_*k*_ indicates the trainable embedding of the *kth* relation; α_*t, i*_ represents the RBA score, which can weigh the importance of each word to the relational expression greatly.

Only under the circumstance that the relation is positive to the sentence do the relation-oriented representations make sense to the following entity extraction. To adaptively control the relation information provided by the previous attention layer, the gated operation mechanism (GOM), which is defined as follows, is applied:


(13) $$\begin{array}{l} g_{k}=\sigma \left(\left[\left(W_{1}M+b_{1}\right);\left(W_{2}c_{k}+b_{2}\right)\right]\right) \end{array}$$

(14) $$\begin{array}{l} u_{k}=g_{k}⊙tanh\left(W_{3}c_{k}+b_{3}\right) \end{array}$$

where *W*_1_, *W*_2_, *W*_3_, *b*_1_, *b*_2_, *and b*_3_ represent learnable parameters, and ⊙ is the dot product. σ indicates the elementwise sigmoid activation function, which returns values from 0 to 1. Therefore, the final results are taken as the percentage of information to maintain. Equation (14) aims to weigh whether the inherent sentence representation *M* or the relation-based representation *c*_*k*_ is more effective for entity extraction; *u*_*k*_ represents the reserved relational feature. The final representation of the *ith* word is obtained by concatenating *m*_*i*_ and *u*_*k*_ as follows:


(15) $$\begin{array}{l} m_{i}^{k}\;=\;\left[m_{i};u_{k}\right] \end{array}$$

The sentence is thus represented as $M^{k}={\left\{m_{i}^{k}\right\}}_{i=\;1}^{n}$.

### Decoder Layer

The decoder part contains the MRC for extracting relations and the MGLSTM for detecting entities. An attention module is applied to match the corresponding entities in line with the identified relations. Finally, the MRT mechanism is introduced to train the model to generate triples. In the following part, the composition of the decoder part will be illustrated in detail.

#### Multiple Relation Classifier

The relation prediction task is a multi-label classification task, aiming to recognize all relation types in text. Commonly, one sentence includes multiple relation triples, which have connections to each other. The MRC is introduced for relations prediction, effectively preventing the phenomenon from happening that multiple classifiers predict the same relation.

To train better relation classifiers to improve the classification accuracy, the output vector *M*^*k*^ of the encoder layer and the global embedding *G* in MGLSTM are fused to constructure the relation layer $\delta \in {\mathbb{R}}^{n\times d_{r}}$.


(16) $$\begin{array}{l} \delta \;=\;Norm\left(\left[G;\;M^{k}\right]\right) \end{array}$$

Then a convolution operator *Conv* and a max-pooling operator are applied on the relation layer δ to generate the text embedding ρ:


(17) $$\begin{array}{l} \varphi \;=\;Conv\left(\delta \right) \end{array}$$

(18) $$\begin{array}{l} \rho =relu\left(maxp\left(\varphi \right)\right) \end{array}$$

where φ ∈ ℝ^*m*×(*n*−*l*+1)^, *m* represents the number of different filters, *n* denotes the length of the text, and *l* indicates the convolutional filter size.

The binary classifier for the *jth* relation type is defined as follows:


(19) $$\begin{array}{l} R_{j}\;=\;\rho W_{H}^{j}+b_{r} \end{array}$$

(20) $$\begin{array}{l} P_{rel}^{j}\left(\hat{R}|S;W_{R}^{j}\right)=softmax\left(R_{j}W_{R}^{j}\right) \end{array}$$

where *R*_*j*_ represents the relation embedding for *jth* relation type, and $P_{rel}^{j}$ denotes the probability distribution that whether the text contains the *jth* relation type or not; $W_{H}^{j}$ and $W_{R}^{j}$ are learnable weight parameters. If the text contains the *jth* relation type, the relation embedding *R*_*j*_ will be fed into the joint extraction module (AJE) to aid entity pair recognition.

The training objective is to minimize the loss function *L*_*rel*_


(21) $$\begin{array}{l} L_{rel}\left(W_{r}\right)\;=\;\frac{1}{\left|S\right|}\overset{\left|s\right|}{\underset{i\;=\;1}{\sum }}\log P_{rel}^{j}\left(\;\hat{R_{j}}{=R}_{j}|S;W_{r};b_{r}\right) \end{array}$$

where *W*_*r*_ represents the weight matrix, *b*_*r*_ denotes the bias vector, $\hat{R_{j}}$ indicates the predicted relation, and *R*_*j*_ is the real relation.

#### MGLSTM

**Figure 4:**
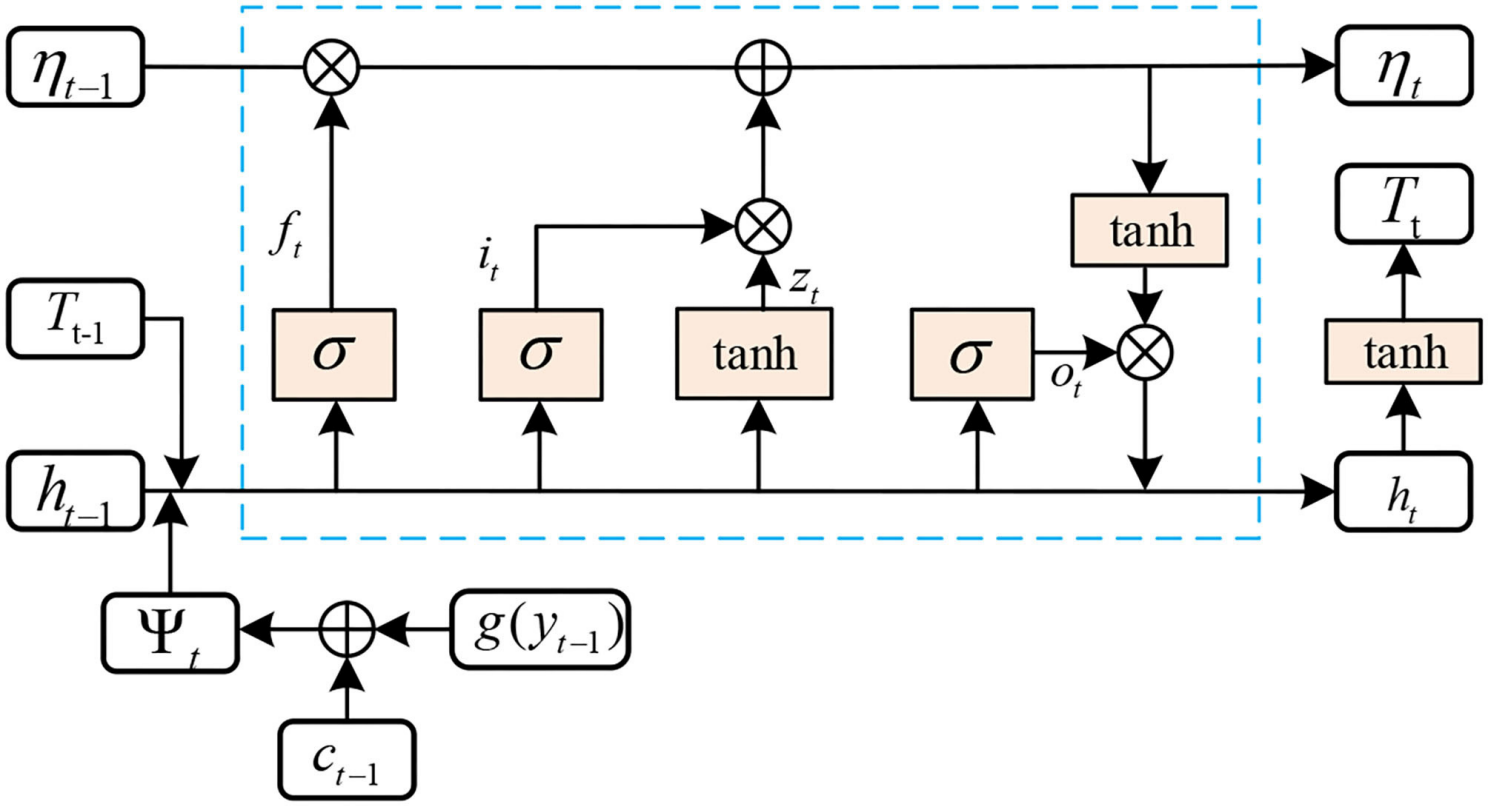
The structure of MGLSTM.

The entity extraction is taken as a sequence labeling task, which focuses on correctly detecting and identifying all entities with relations in one sentence *S*. During this process, we do not determine the relation type between two entities. To realize this objective, the MGLSTM is applied, as shown in Figure 4, which is an excellent variant of LSTM. It inherits the most characteristics of RNN models and solves the problem of gradient disappearance in the gradient backpropagation process. The detailed operations are defined as follows:


(22) $$\begin{array}{l} \psi_{t}\;=\;\left[g\left(y_{t\;-\;1}\right);c_{t\;-\;1}\right] \end{array}$$

(23) $$\begin{array}{l} i_{t}\;=\;\sigma \left(W_{wi}\psi_{t}\;+\;W_{hi}h_{t\;-\;1}\;+\;W_{ti}T_{t\;-\;1}\;+\;b_{i}\right) \end{array}$$

(24) $$\begin{array}{l} f_{t}\;=\;\sigma \left(W_{wf}\psi_{t}\;+\;W_{hf}h_{t\;-\;1}\;+\;W_{tf}T_{t\;-\;1}\;+\;b_{f}\right) \end{array}$$

(25) $$\begin{array}{l} z_{t}\;=\;\tanh\left(W_{wc}\psi_{t}\;+\;W_{hc}h_{t\;-\;1}\;+\;W_{tc}T_{t\;-\;1}\;+\;b_{c}\right) \end{array}$$

(26) $$\begin{array}{l} \eta_{t}\;=\;f_{t}\eta_{t\;-\;1}\;+\;i_{t}z_{t} \end{array}$$

(27) $$\begin{array}{l} o_{t}\;=\;\sigma \left(W_{wo}\psi_{t}\;+\;W_{ho}h_{t\;-\;1}\;+\;W_{c0}\eta_{t}\;+\;b_{0}\right) \end{array}$$

(28) $$\begin{array}{l} h_{t}\;=\;o_{t}\tanh\left(\eta_{t}\right) \end{array}$$

(29) $$\begin{array}{l} T_{t}\;=\;\tanh\left(h_{t}\right) \end{array}$$

where $\psi ={\left\{\psi_{t}\right\}}_{t=1}^{n}$ denotes the input, *g*(*y*_*t*−1_) indicates the global embedding, *c*_*t*−1_ represents attention context at time step *t* − 1, and σ denotes the logistic sigmoid function. In detail, the forget gate *f*_*t*_controls how much the previous memory cell is forgotten, the input gate *i*_*t*_ controls how much information is input to each unit, and the output gate *o*_*t*_controls the exposure of the internal memory state. $T={\left\{T_{t}\right\}}_{t=1}^{n}$ denotes the output which can predict all entities in the text.

Commonly, the predicted labels in the previous steps have some influence on the label prediction in the following steps. This is a classical exposure bias phenomenon, which might occur in all time steps. To overcome this challenge, all information at the time step *t* − 1 should be considered. Therefore, the global embedding *g*(*y*_*t*−1_) is introduced, which represents the information of all candidate labels that exist in *y*_*t*−1_ at the (*t*−1)th time step; *y*_*t*−1_ represents the predicted probability distribution at time step *t* − 1. The global embedding *g*(*y*_*t*−1_) is calculated as follows:


(30) $$\begin{array}{l} ē\;=\;\overset{n}{\underset{i\;=\;1}{\sum }}y_{t\;-\;1}^{\left(i\right)}e_{i} \end{array}$$

(31) $$\gamma \;=\;\sigma (W_{1}e\;+\;W_{2}\bar{e})$$

(32) $$\begin{array}{l} g\left(y_{t\;-\;1}\right)\;=\;\left(1\;-\;\gamma \right)⊙e\;+\;\gamma ⊙ē \end{array}$$

where *e* represents the embedding of the label which has the biggest probability under the distribution *y*_*t*−1_. denotes the weighted average embedding at time *t*; $y_{t-1}^{\left(i\right)}$represents the *i*th element of *y*_*t*−1_, and *e*_*i*_ denotes the embedding vector of the *i*th label; γ denotes the transform gate used for regulating the proportion of the weighted average embedding. Global embedding *g*(*y*_*t*−1_) is the optimized combination of the original embedding and the weighted average embedding with transformed gate γ. The global embedding improves the performance of the model significantly, since the source information is optimized when predicting the label at time step *t*.

In addition, the label sequence of each sample is sorted based on the frequency of labels in the training set. To predict the tag ${\hat{Tag}}_{i}$, a Softmax layer is applied to get the final probability distribution *P*_*ent*_ over the label space ϑ, which is computed as follows.


(33) $$\begin{array}{l} \vartheta \;=\;W_{o}\varphi \left(W_{h}h\;+\;W_{c}c\right) \end{array}$$

(34) $$\begin{array}{l} P_{ent}\left({\hat{Tag}}_{i}|\text{S};{\text{W}}_{s}\right)\;=\;softmax\left(\left(\vartheta \;+\;{\text{I}}_{t}\right){\text{W}}_{sf}+{\text{b}}_{sf}\right) \end{array}$$

(35) $${\left(I_{t}\right)}_{i}\;=\{\begin{array}{c} \;-\;\infty \;if\;the\;label\;l_{i}\;has\;been\;predicted\;at\;previous\;t-1\;time\;steps\\ 0\;otherwise \end{array}$$

where *W*_*o*_, *W*_*h*_, *W*_*c*_, and *W*_*sf*_ denote the weight matrices, and *b*_*sf*_ represents the bias vector; *I*_*t*_ is the mask vector which aims to prevent the decoder from generating repeated labels; φ represents a non-linear activation function.

In the training process, the cross-entropy loss is taken as the loss function, and the beam search algorithm (Wiseman and Rush, 2016) is applied to figure out the top-ranked prediction path. Given an input sentence *S* and its ground-truth tag sequence *Tag*, the training objective is to minimize loss function *L*_*ent*_.


(36) $$\begin{array}{l} L_{ent}\left(W_{all}\right)\;=\;-\;\frac{1}{\left|s\right|}\overset{\left|s\right|}{\underset{i\;=\;1}{\sum }}\log P_{ent}\left({{\hat{Tag}}_{i}\;=\;Tag}_{i}|S;W_{all};b_{all}\right) \end{array}$$

where *W*_*all*_ denotes weight matrices, *b*_*all*_ represents bias vector, $\hat{Tag}$ indicates the predicted tag, and *Tag* stands for the real tag.

#### Joint Extraction of Entities and Relations

After detecting all relations and all entities, the extraction of triples is realized by applying the AJE. During this process, two entities are selected as the target entity pair, and the different entity pairs are assigned the different target relations. The weighted value generated by the attention mechanism represents the matching degree between the token and the target relation type. To get the attention weight of the *ith* predicted relation, the annotation sequence $\beta_{t}^{i}$ of the *ith* relation at time step *t* is calculated as follows:


(37) $$\begin{array}{l} \beta_{t}^{i}\;=\;\tanh\left(W^{r}o^{ci}\;+\;W^{d}G_{t\;-\;1}\;+\;W^{p}m_{i}\right) \end{array}$$

where *o*^*ci*^ indicates the trainable embedding of the *ith* relation. *G*_*t*−1_ denotes the global embedding generated by the entity prediction at time step *t* − 1; *m*_*i*_ represents the embedding generated by the encoder. *W*^*r*^, *W*^*d*^, *and W*^*p*^ represent learnable parameters.

The attention distribution α_*t*_ on the annotation sequence β_*i*_ of text is computed as follows:


(38) $$\begin{array}{l} a_{t}^{i}\;=\;sofmax\left(\beta_{i}\right) \end{array}$$

where $\alpha_{t}={\left\{\alpha_{t}^{i}\right\}}_{i=1}^{n}$, *n* denotes the text length. $a_{t}^{i}$ denotes the output at time step *t*.

Finally, the label sequence $\varepsilon^{i}={\left\{\varepsilon_{t}^{i}\right\}}_{t=1}^{n}$ corresponding to the *ith* relation is calculated by follows:


(39) $$\begin{array}{l} \rho_{i}^{t}\;=\;\overset{n}{\underset{i\;=\;1}{\sum }}a_{t}^{i}\;\times l_{t}^{i} \end{array}$$

(40) $$\begin{array}{l} \varepsilon_{t}^{i}\;=\;sofmax\left(\rho_{t}^{i}\right) \end{array}$$

where $l_{t}^{i}$ denotes the input vector at time step *t* and $\rho_{i}^{t}$ represents the context vector. The sequence vector generated for the *ith* relation is $\rho_{i}={\left\{\rho_{i}^{t}\right\}}_{t=\;1}^{n}$.

### Training

#### The MRT Framework

To make the training more stable, the MRT framework is introduced. We pretrain the model with local loss in the first step, and we optimize the local loss *L*_*local*_ and the global loss *L*_*mrt*_ simultaneously in the second step. The local loss *L*_*local*_, defined in Equation (41), is the linear combination of the MRC loss *L*_*rel*_ and the MGLSTM loss*L*_*ent*_:


(41) $$\begin{array}{l} L_{local}=\lambda \cdot L_{rel}+\left(1-\lambda \right)\cdot L_{ent} \end{array}$$

where λ is a hyperparameter to balance the RC task and the NER task.

The global loss *L*_*mrt*_ provides a tighter connection between the entity extraction task and the relation classification task. To illustrate the algorithm, we first aggregate some notations. Let *y* ≜ (*Tag, R*) contain the ground truth entity tag sequence and relations, $ŷ≜\left(\hat{Tag},\;\hat{R}\right)$ contain the predicted entity tag sequence and relations, and ∅(*S*) be the set of all possible outputs of the input sentence *S* [*y*, ŷ ∈ ∅(*S*)]. We define the joint probability by


(42) $$\begin{array}{l} P\left(ŷ|S;W\right)=P\left(\hat{Tag}|S,Tag;W_{st}\right)P\left(\hat{R}|S,R;W_{R}\right) \end{array}$$

where *W* = *W*_*st*_ ∪ *W*_*R*_ represents the set of the model parameters. The MRT loss *L*_*mrt*_ is defined by


(43) $$Q\left(\hat{y}|S;W,\;u,\;\alpha_{mr}\right)\;=\;\frac{1}{z}{\left[P{\left(\hat{Tag}|S,\;W_{st}\right)}^{u}P{\left(\hat{R_{i}}|S,\;\hat{Tag},\;W_{R}\right)}^{1\;-\;u}\right]}^{\alpha_{mr}}$$

(44) $$Z\;=\;\sum_{\left(Tag^{\prime },\;R^{\prime })\in \emptyset^{\prime }(S\right)}{\left[P{\left(Tag^{\prime }|S,\;W_{st}\right)}^{u},\;Tag^{\prime },\;W_{R}\right)}^{1\;-\;u}{]}^{\alpha_{mr}}$$

(45) $$\Delta (y,\hat{y})=\frac{1}{n_{tri}}\sum_{i}-[y_{i}\cdot log(P(\hat{y}|S;W))+(1-y_{i})\cdot log(1-P(\hat{y}|S;W))]$$

(46) $$\begin{array}{l} L_{mrt}\left(W\right)=\underset{ŷ\in \emptyset^{\prime }\left(S\right)}{\sum }Q\left(ŷ|S;W,\;u,\;\alpha_{mr}\right)\Delta \left(y,\;ŷ\right) \end{array}$$

where *Q*(ŷ|*S*; *W, u*, α_*mr*_) is a re-normalization of *P*(ŷ|*S*; *W*) on the subset ∅′(*S*). The sharpness of *Q* distribution (Och, 2017) is regulated by hyperparameter α_*mr*_, and *u* measures the significance of the entity model and the relation model in *Q*; *n*_*tri*_ represents the number of triples.

#### Training Algorithm

The complete training step for ROMGCJE is summarized in Algorithm 1. The embedding dimensions, learning rate, dilation rates, the number of heads, and so on., will be initialized before training. During the training process, the mini-batch samples are first fed into the encoder layer, which contains PTSRL, CWRL, and RBAGM. Then we obtain the hidden states $M^{k}={\left\{m_{t}^{k}\right\}}_{t=1}^{n}$ of the encoder, which contains rich contextual information and semantic information. Next, the MRC and MGLSTM in the decoder layer are applied to complete the NER and RC tasks. After the detection of all relations and all entities, the label sequences of triples $\varepsilon^{i}={\left\{\varepsilon_{t}^{i}\right\}}_{t=1}^{n}$ are realized by the application of the AJE. Finally, the whole model is trained by the MRT framework, which updates all parameters based on the mini-batch gradient descent.

**Algorithm 1:**
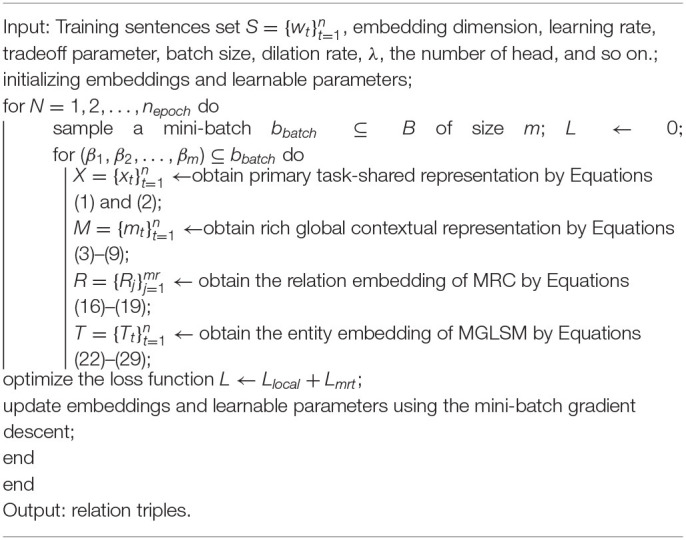
Training algorithm for ROMGCJE.

## Experiments

To evaluate the effectiveness of the new model, extensive experiments are conducted on the two different public datasets NYT and WebNLG.

### Dataset

The NYT dataset was obtained based on the distant supervision method without artificial labeling (Riedel et al., 2010). WebNLG dataset was introduced by Gardent et al. (2017) for the natural language generation task and later was applied to the triple extraction task. The summary statistics of the two datasets are shown in Table 2. The test set is divided into different groups according to the triple overlapping degree and the number of triples in one sentence.

**Table 2:** Statistics of datasets.

**Dataset**	**Train**	**Valid**	**Test**	**Triple overlapping degree**			**Number of triples**					**Rel**.
				**Normal**	**SEO**	**EPO**	**=1**	**=2**	**=3**	**=4**	**≥5**	
NYT	56,195	5,000	5,000	3,266	1,297	978	3,244	1,045	312	291	108	24
WebNLG	5,019	500	703	246	457	26	266	171	131	90	45	216^*^

* *Note that the right number of relations in WebNLG dataset is 216, but it was miswritten as 246 in (Zeng et al., 2018)*.

### Hyperparameters

Our experiments are conducted under the environment of Python3.7 +Theano + Cuda10.0. The dimension of BERT is initialized as 128. The window size of BiLSTM is set to 3, the number of filters is 50, and the following dense layer has a hidden layer with 100 dimensions. For MDiconv, the dilation rates are 1, 2, and 4. The embedding and classification layers are standardized by dropout with a ratio of 0.5. For Multi-head attention, the head number is set to 8. The dimension of the MGLSTM is set as 256, and the cell unit number is set as 100. The learning rate is set to 0.001, and the batch size is 64. The mini-batch gradient descent is applied to optimize parameters.

### Baselines

To evaluate the effectiveness of this new model, extensive contrast experiments are carried out with the following state-of-the-art triple extraction models:

(1) Novel tagging (Zheng et al., 2017): This model converts the joint extraction task to a sequential labeling problem by a tagging scheme where each token is assigned a unique tag denoting entity mentions and relation types simultaneously.

(2) CopyRE (Zeng et al., 2018): This RE model is an end-to-end neural model with a copy mechanism. The principle is that the relation is first extracted, and then the corresponding entity pair is extracted by a copy mechanism from the source texts.

(3) GraphRel (Fu and Ma, 2019): Based on GCNs, this end-to-end joint extraction model can predict relations between all word pairs. It constructs a complete word graph for each sentence accurately.

(4) CopyMTL (Zeng et al., 2020): Based on a multi-task learning framework equipped with a copy mechanism, CopyMTL is constructed to predict multi-token entities.

(5) HRL (Takanobu et al., 2019): This model applies a hierarchical reinforcement learning framework that decomposes the task into a high-level task for relation detection and a low-level task for entity extraction.

(6) RSAN (Yuan et al., 2020): The relation-aware attention mechanism is applied in RSAN to construct specific sentence representations for each relation. Then the corresponding head and tail entities are extracted by performing the sequence labeling.

(7) WDec (Nayak and Ng, 2020): A novel triples representation scheme is proposed, and the sequence-to-sequence mechanism is employed to produce the word sequences.

(8) TPLinker (Wang et al., 2020a): TPLinker treats the joint extraction task as a token pair linking problem to overcome the overlapping triple challenge. There are no interdependent stages; thus, the error accumulation is alleviated.

### Performance Metrics

A relational triple is considered correct, where the two entities and the corresponding relation type are all correct. The standard Precision (Prec.), Recall (Rec.), and F1 scores are selected as the evaluation matrix of experiment results.

### Experimental Results

#### Comparative Experiments

To show the effectiveness of the new model ROMGCJE in extracting triples, we first carry out the plenty of contrast experiments with some state-of-the-art methods on public datasets NYT and WebNLG. The test results are presented in Table 3, and the best experiment results are denoted by the bold numbers. It is easy to conclude that the performances of other existing models do not surpass our model on both two datasets. This new model achieves a 1.6 and 1.0% gain in F1 over the best method TPLinker on datasets NYT and WebNLG, respectively. The following points might contribute to the improvement of ROMGCJE: (1) The sufficient contextual information and long-range dependency captured by PTSRL and CWRL benefit NER and RC tasks greatly. (2) The MGLSTM can capture sufficient global information. The global embedding from MGLSTM contributes to the RC task, which excludes the error caused by predictions of the wrong relationship.

**Table 3:** Comparison results of ROMGCJE with other models.

**Methods**	**NYT**			**WebNLG**		
	**Prec**.	**Rec**.	**F1**	**Prec**.	**Rec**.	**F1**
Noveltagging	0.621	0.312	0.424	0.516	0.185	0.276
CopyRE	0.608	0.570	0.580	0.370	0.356	0.361
GraphRel	0.631	0.596	0.611	0.406	0.402	0.419
CopyMTL	0.750	0.675	0.716	0.572	0.539	0.556
HRL	0.769	0.768	0.762	–	–	–
RSAN	0.855	0.825	0.832	0.796	0.828	0.814
WDec	0.875	0.754	0.805	0.835	0.630	0.725
TPLinker	0.895	0.931	0.932	0.853	0.920	0.939
ROMGCJE	**0.910**	**0.939**	**0.948**	**0.905**	**0.938**	**0.949**

*The best experiment results are denoted by the bold numbers*.

#### Performance on Normal, EPO, and SEO Sentences

**Figure 5:**
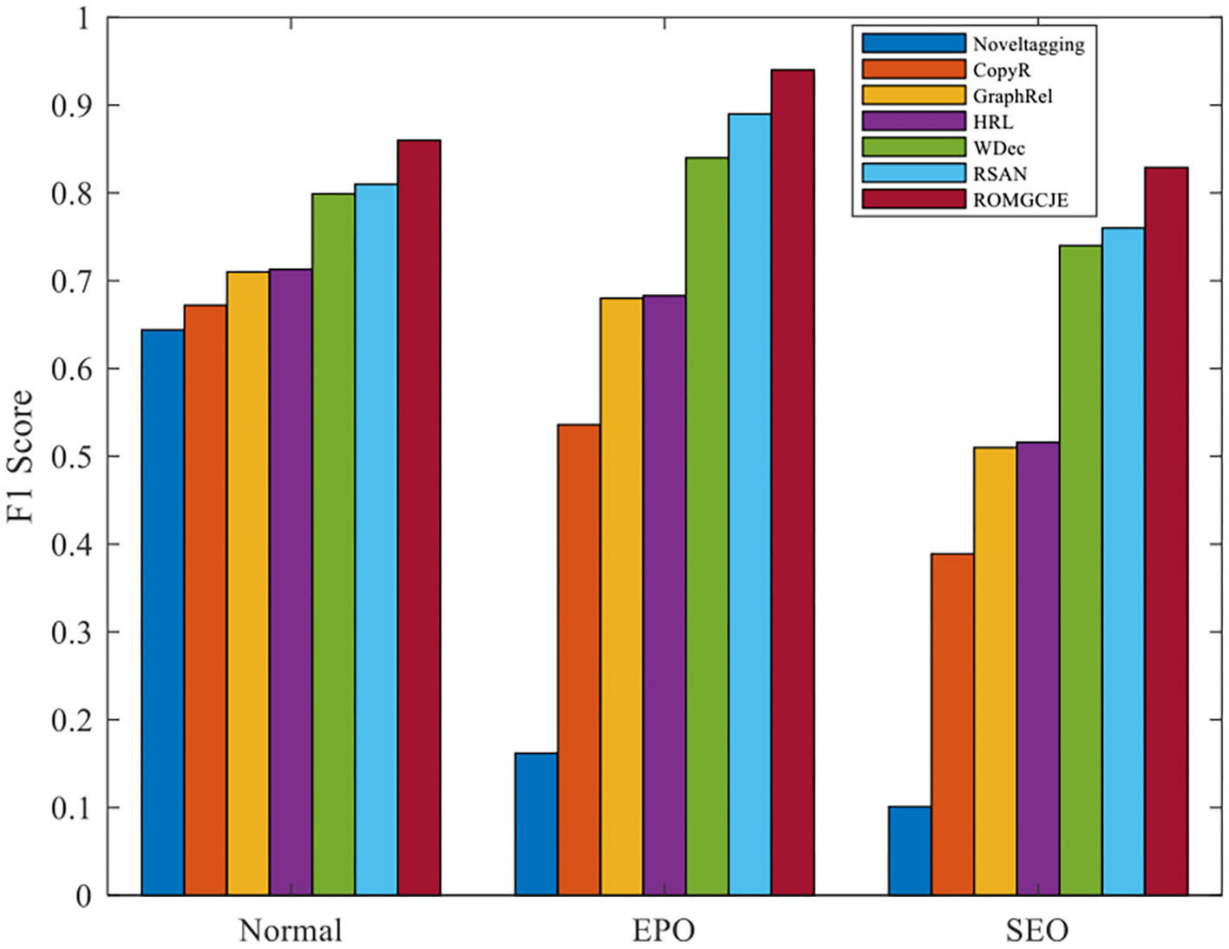
Comparative experiments on normal, EPO, and SEO sentences.

To further explore the capability of ROMGCJE in extracting overlapping relations, extensive experiments are developed on the NYT dataset. Based on the triple overlapping degree, the NYT test set is divided into the following three sub-sets: Normal sentence set, SEO sentence set, and EPO sentence set. Figure 5 shows the F1 values of the different comparative models on NYT test data. The different color blocks denote the experiment results of the different models. The F1 score of ROMGCJE surpasses other models on normal, EPO, and SEO sentences. In particular, ROMGCJE shows more outcoming performance on the EPO sentences than other comparative models. There are three reasons for this performance as follows: (1) The new labeling scheme can assign different tags to a word, (2) the new model ROMGCJE can make separate predictions for the different relations, and (3) the two attention modules have some contributions to the prediction of EPO triples.

In the following parts, we conduct more ablation studies to verify the effectiveness of each module in ROMGCJE and further explore the reasons for the great improvement.

#### Ablation for Character Embedding

To evaluate the contribution of the character representation captured by BiLSTM in the PTSRL, some ablation tests are conducted on NYT and WebNLG datasets. The experiment results are shown in Table 4. “–Bidirectional long short-term memory neural network” means that the character embedding is not used in PTSRL, and the word representation learned by BERT is directly fed to CWRL. From Table 4, we can see that the introduction of character embedding promotes a 1.6% increment of F1. We attribute this phenomenon to that the character embedding includes the local feature of input text and greatly contributes to extracting the morphological information and dealing with the out-of-vocabulary problem.

**Table 4:** Ablation studies of ROMGCJE on NYT and WebNLG dataset.

**Methods**	**NYT**			**WebNLG**		
	**Prec**.	**Rec**.	**F1**	**Prec**.	**Rec**.	**F1**
ROMGCJE	0.910	0.939	0.948	0.905	0.938	0.949
–BiLSTM	0.901	0.930	0.932	0.897	0.930	0.941
–RBAGM	0.879	0.908	0.913	0.861	0.903	0.915
–AJE	0.856	0.881	0.897	0.865	0.898	0.901

#### Ablation for Attention Module

To demonstrate the contribution of the two attention modules, RBAGM and AJE, plenty of ablation tests are conducted on the NYT dataset. The experimental results are summarized in Table 4. “—RBAGM” denotes that the RBAGM module is not applied when extracting triples. Similarly, “–AJE” means that the AJE module is not applied in GCRE. As we can see from Table 4, the model's Precision drops significantly when RBAGM or AJE is deleted from ROMGCJE. We can conclude that the sentence representations fused with the fine-grained semantic relation feature greatly affect the joint extraction task.

**Figure 6:**
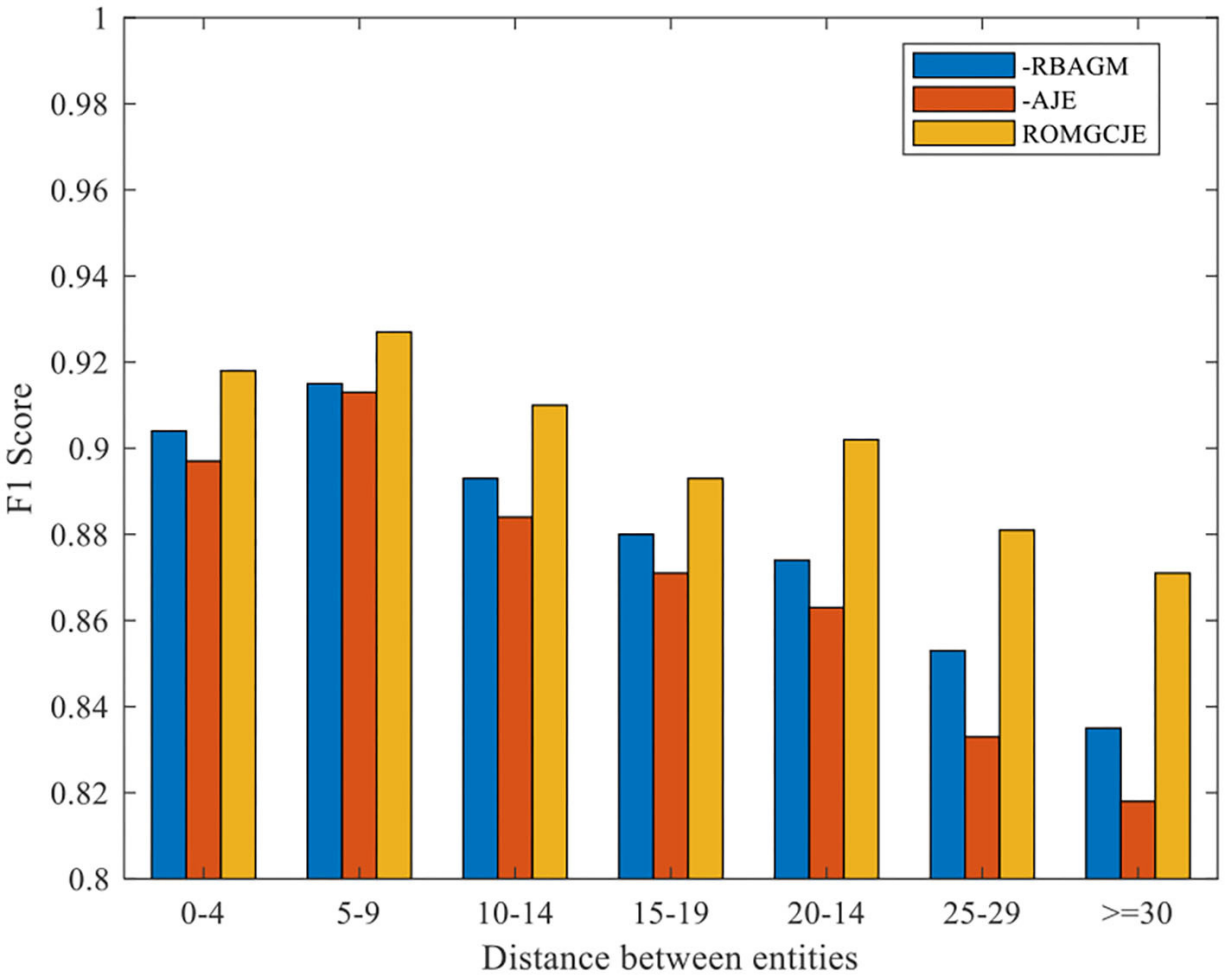
Comparison of ROMGCJE, “–RBAGM,” and “–AJE” under different entity distances on the NYT dataset.

**Figure 7:**
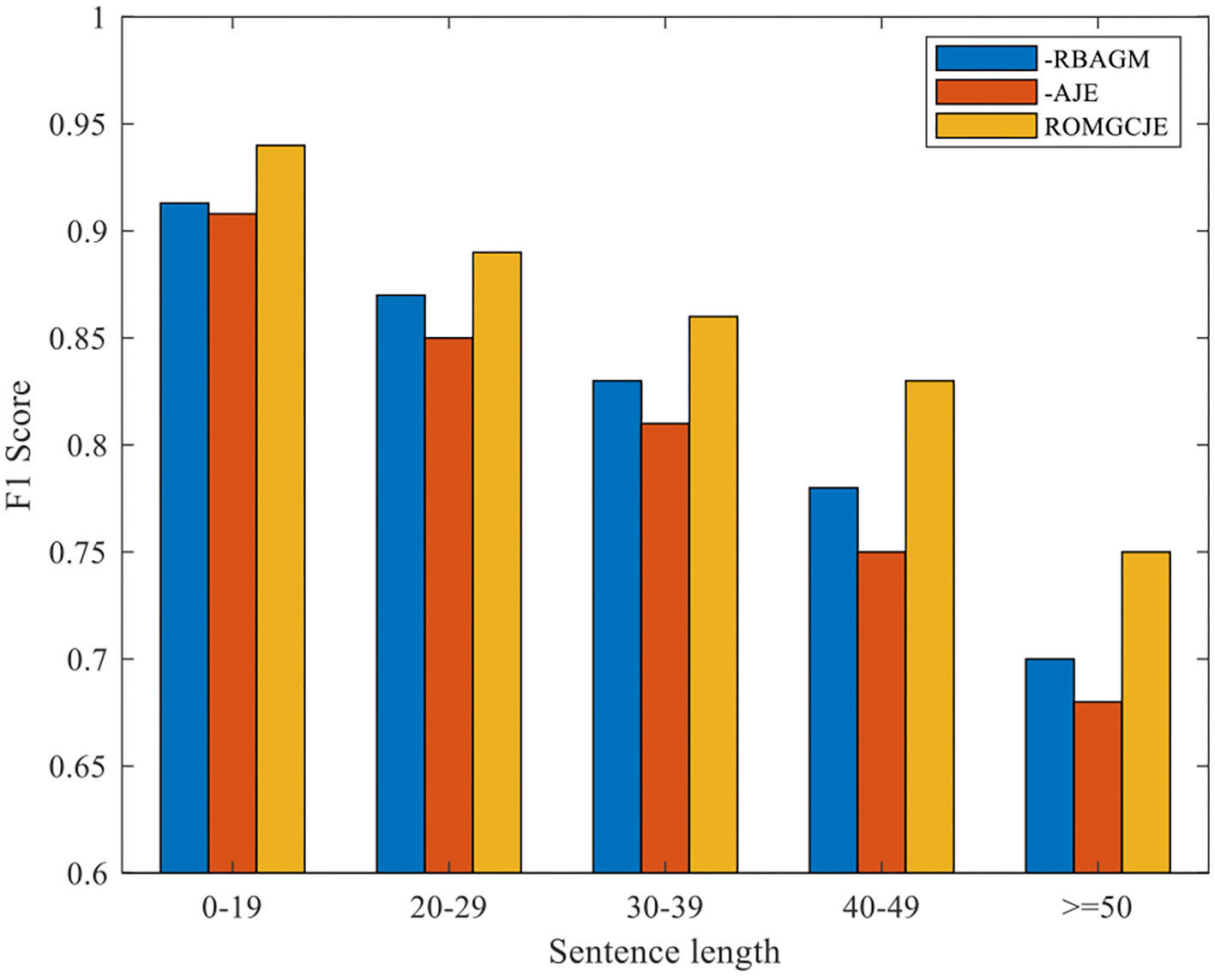
Comparison of the ROMGCJE, “–RBAGM,” and “–AJE” under different sentence lengths on the NYT dataset.

Besides, we conduct the following two groups of experiments on NYT dataset: (1) Exploring the influence of the different distances between entities on ROMGCJE and (2) exploring the influence of sentence length on ROMGCJE. The experimental results are presented in Figures 6, 7, respectively, weighted by the F1 score. The entity distance is measured by the rule that the absolute character offset between the last character of the first occurring entity and the last character of the second-mentioned entity. From Figure 6 we can conclude that ROMGCJE significantly outperforms “–RBAGM” and “–AJE” across the different entity distances. The introduction of AJE and RBAGM makes the model ROMGCJE increase by 4.1 and 3.4% in F1 score, respectively, when the entity distance is more than 20 characters. It is easy to conclude that AJE and RBAGM contribute greatly to the triple extraction task. In the second group of experiments, we partition the NYT dataset into five groups based on the sentence length [(0–19), (20–29), (30–39), (40–49), (≥50)]. We analyze the performance of ROMGCJE, “–RBAGM,” and “–AJE” on these five subsets, as shown in Figure 7. We can observe a decline in the F1 score of these three models when sentences contain more words. However, the performance of ROMGCJE still outperforms that of “–RBAGM” and “–AJE.” Moreover, ROMGCJE outperforms “–RBAGM” and “–AJE” by 5.26 and 6.42% in the F1 score for triple extraction, respectively, when the sentence contains more than 40 words.

We analyze the above results from the following two perspectives: (1) For AJE, it promotes the interaction between RC and NER tasks since it can pass entity information from NER task to RC task and collect RC task's feedback information by jointly updating all the parameters. (2) For RBAGM, it plays a significant role in capturing the global dependencies of the whole sentence, which greatly contributes to the prediction of EPO and SEO triples in long sentences. In EPO sentences, the relation is different while the entity pairs are the same; thus, these entities have more sufficient semantic information, which attracts more attention in decoding. Finally, it is more possible for these entities to be selected from the input sentence. Based on the above analysis, the model ROMGCJE has great advantages in extracting entities and relations from long sentences with EPO or SEO triples.

#### Ablation for CWRL Module

Plenty of ablation experiments are conducted on the NYT dataset to find the best combination of MHAttention and MDiconv. The experimental results are presented in Table 5. When the number of both MHAttention and MDiconv is set to 0, the PTSRL is directly applied to encode tokens without applying the max-pooling module. We can observe that the Prec., Rec., F1 scores drop of 13.8, 14.4, and 13.1%, respectively, indicating that CWRL is critical for improving model performance. Once the number of MHAttention or MDiconv increases, the model's performance improves to a different extent. This suggests that the contextual information obtained by CWRL can greatly assist ROMGCJE in jointly extracting entities and relations. After analyzing the prediction results of the model with different combinations of MHAttention and MDiconv, we conclude that the number of MHAttention and MDiconv is set to 2 and 2, respectively, making the ROMGCJE model achieve the best performance. Further, with the number of Mdiconv or MHAttention increasing from 1 to 5, the values of the evaluation matrix increase first but decrease later. Thus, we can obtain that more Mdiconv modules or MHAttention modules are not always better.

**Table 5:** Ablation experiments for CWRL with different combinations.

**Multi-head Attention**	**MDiconv**	**Prec**.	**Rec**.	**F1**
0	0	0.772	0.795	0.817
0	1	0.791	0.815	0.822
0	2	0.811	0.834	0.848
0	3	0.835	0.881	0.890
0	4	0.857	0.893	0.901
1	0	0.752	0.789	0.790
1	1	0.768	0.801	0.801
1	2	0.820	0.853	0.859
1	3	0.854	0.883	0.889
1	4	0.836	0.865	0.879
2	0	0.844	0.873	0.889
2	1	0.887	0.901	0.912
2	2	**0.910**	**0.939**	**0.948**
2	3	0.891	0.921	0.923
2	4	0.853	0.889	0.897
3	0	0.790	0.831	0.830
3	1	0.821	0.851	0.857
3	2	0.864	0.893	0.896
3	3	0.831	0.861	0.879
3	4	0.801	0.841	0.843
4	0	0.756	0.783	0.789
4	1	0.813	0.843	0.850
4	2	0.806	0.838	0.845
4	3	0.798	0.831	0.839
4	4	0.768	0.809	0.812

*The best experiment results are denoted by the bold numbers*.

#### Ablation for MGLSTM

**Figure 8:**
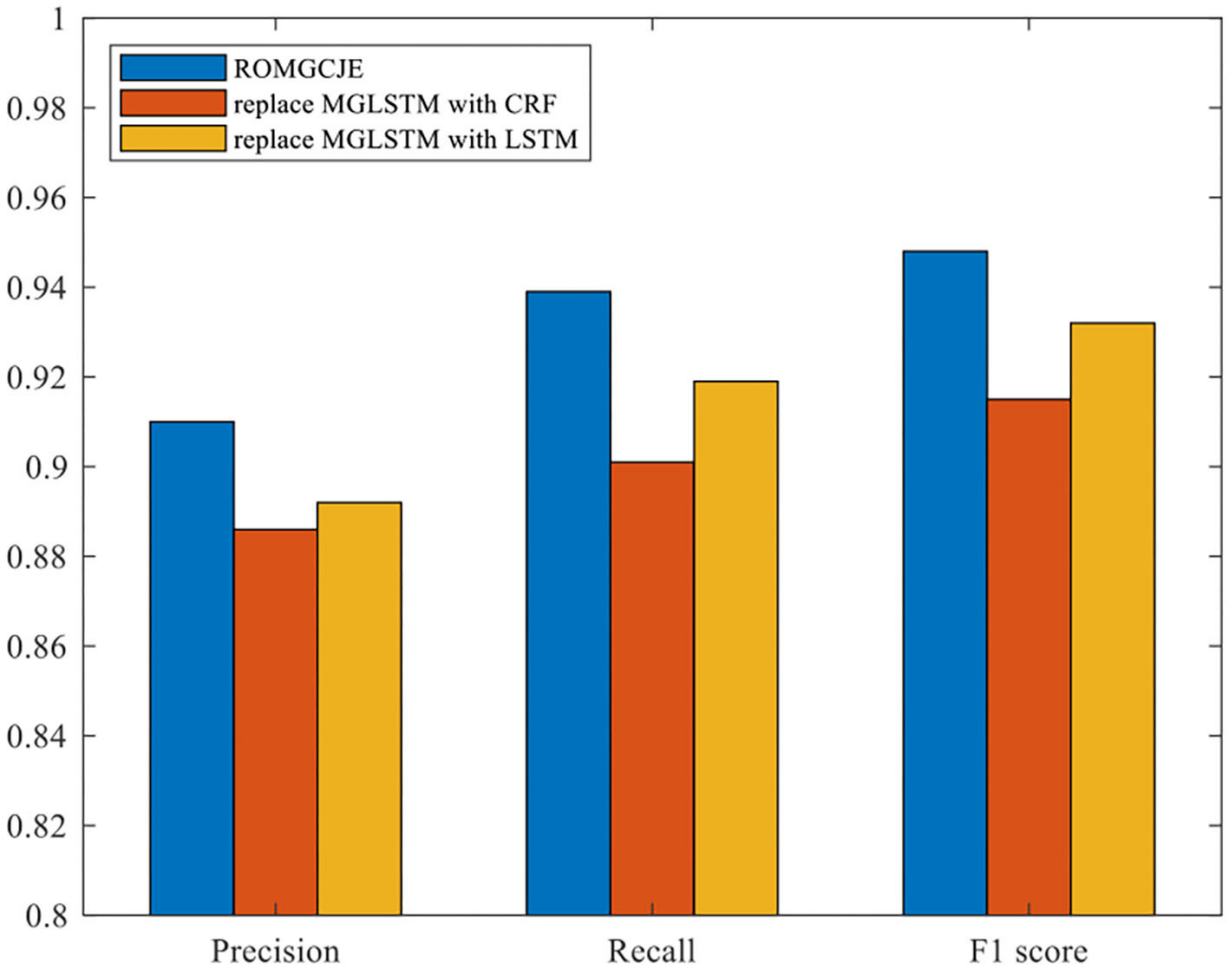
Ablation experiments for MGLSTM on the NYT dataset.

Plenty of ablation experiments are conducted on the NYT dataset to explore the effects of MGLSTM for NER in the decoder part. In this process, the MGLSTM is replaced by CRF and LSTM, respectively, and the final results are presented in Figure 8. Also, “replace MGLSTM with CRF” means that the MGLSTM is replaced by the CRF module in ROMGCJE; “replace MGLSTM with LSTM” refers to that LSTM replaces the MGLSTM. From Figure 8, we can see that the MGLSTM for NER makes the model achieve the best performance. Using CRF for NER leads to a reduction of 2.4% Precision in NYT. The reason is that there is a long distance among these relation tags, but CRF has difficulties in overcoming this problem. In contrast, the performance of LSTM is a little better than CRF. It only obtains a reduction of 1.8% Precision in NYT. This is because LSTM can build a long-range dependency to some extent. Besides the merits inherited from LSTM, MGLSTM can learn to represent information over multiple time scales, and the introduction of global embedding can reduce the damage caused by mispredictions made in the previous time steps. Therefore, the application of MGLSTM makes the model predict label sequences more accurately.

#### Ablation Study for MRC

**Figure 9:**
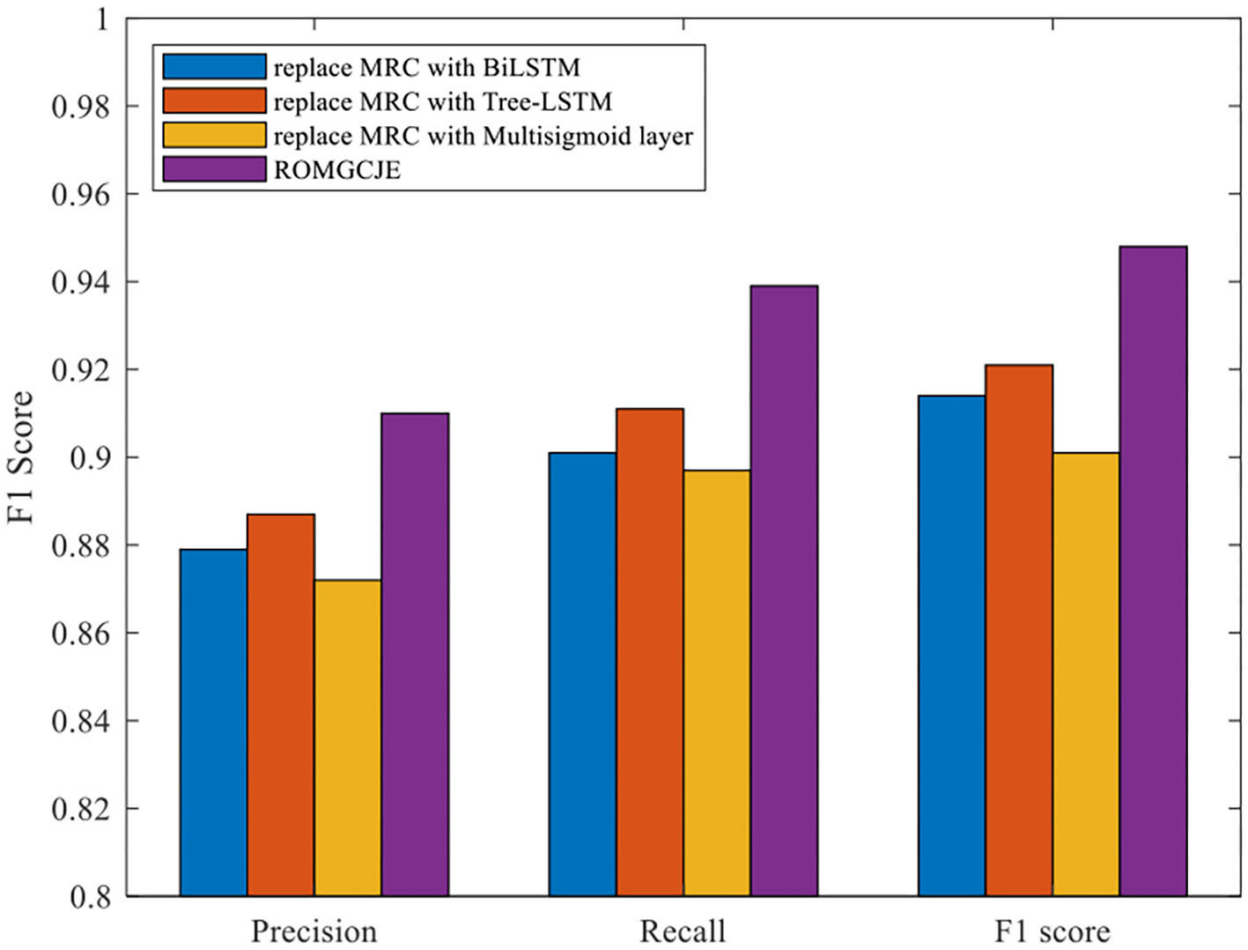
Ablation experiments for MRC on the NYT dataset.

To demonstrate the effectiveness of the MRC module in ROMGCJE, some experiments are developed under the circumstances that these entities are known. The comparison performance on the NYT dataset is presented in Figure 9. There are some different combinations as follows: (1) “replace MRC with BiLSTM” means that the MRC is replaced by BiLSTM (2) “replace MRC with Tree-LSTM” refers to that the MRC is replaced by Tree-LSTM (3) “replace MRC with Multisigmoid layer” represents that the MRC is replaced by Multisigmoid layer. From Figure 9, we can see that ROMGCJE achieves the best result in terms of triples extraction. The possible reason is that BiLSTM, Tree-LSTM, and Multisigmoid layer module have great difficulties in assigning multiple tags to one word, and thus they cannot deal with the overlapping relation problems. The above results demonstrate that the MRC module is very suitable for ROMGCJE. NYT contains much noise data, which indicates that ROMGCJE is robust.

## Conclusions

This study proposes a novel joint extraction model ROMGCJE for overlapping relationships and entities. The introduction of CWRL enables ROMGCJE to capture rich global contextual information, build long-range dependency among words, and fully extract the semantics of the text. Besides, the global embeddings learned by MGLSTM boost the extraction of entities and reduce the error propagation from NER task to RC task. In addition, applying the attention mechanism contributes to the prediction of overlapping relation triples greatly. Comprehensive experiments prove that the proposed method achieves the state-of-the-art performance compared with other approaches.

Based on the model ROMGCJE, one self-learning KG can be developed, which has the ability to better organize, manage and understand the massive information on the Internet. In the future, this self-learning KG can be applied into many fields, such as search engines, intelligent question answering, intelligent recommendation, intelligent furniture, fault diagnosis, and so on. The ROMGCJE model will contribute to the research of the above-mentioned fields significantly.

## Data Availability Statement

The original contributions presented in the study are included in the article/supplementary material, further inquiries can be directed to the corresponding author/s.

## Author Contributions

HH: writing—original draft preparation, writing—reviewing and editing, conceptualization, validation, formal analysis, and methodology. JW: conceptualization, data curation, supervision, project administration, funding acquisition, and resources. XW: supervision, software, validation, formal analysis, investigation, reviewing and editing, and data curation. All authors contributed to the article and approved the submitted version.

## Funding

This work was supported by the National Science and Technology Innovation 2030 of China Next-Generation Artificial Intelligence Major Project, Data-Driven Tripartite Collaborative Decision-Making and Optimization, under Grant 2018AAA0101801.

## Conflict of Interest

The authors declare that the research was conducted in the absence of any commercial or financial relationships that could be construed as a potential conflict of interest.

## Publisher's Note

All claims expressed in this article are solely those of the authors and do not necessarily represent those of their affiliated organizations, or those of the publisher, the editors and the reviewers. Any product that may be evaluated in this article, or claim that may be made by its manufacturer, is not guaranteed or endorsed by the publisher.
